# CFD Studies on Biomass Thermochemical Conversion

**DOI:** 10.3390/ijms9061108

**Published:** 2008-06-27

**Authors:** Yiqun Wang, Lifeng Yan

**Affiliations:** Department of Chemical Physics and Hefei National Laboratory for Physical Science at the Microscale, University of Science and Technology of China, Hefei, 230026, P. R. China

**Keywords:** Biomass, CFD, thermochemical, gasification, pyrolysis, combustion, model

## Abstract

Thermochemical conversion of biomass offers an efficient and economically process to provide gaseous, liquid and solid fuels and prepare chemicals derived from biomass. Computational fluid dynamic (CFD) modeling applications on biomass thermochemical processes help to optimize the design and operation of thermochemical reactors. Recent progression in numerical techniques and computing efficacy has advanced CFD as a widely used approach to provide efficient design solutions in industry. This paper introduces the fundamentals involved in developing a CFD solution. Mathematical equations governing the fluid flow, heat and mass transfer and chemical reactions in thermochemical systems are described and sub-models for individual processes are presented. It provides a review of various applications of CFD in the biomass thermochemical process field.

## 1. Introduction

The use of biomass as a CO_2_-neutral renewable fuel is becoming more important due to the decreasing resources of fossil fuel and their effect on global warming. Thermochemical conversion of biomass offers a possible process to provide gaseous, liquid and solid fuels and prepare chemicals derived from biomass. Many efforts have been done on making thermochemical processes more efficient and economically acceptable. A significant portion of these efforts over the past two decades has focused on the development of numerical models of thermochemical reactors (such as gasifiers, pyrolyzers, boilers, combustors, incinerators) that can help to design and analyze the thermochemical process. Due to a combination of increased computer efficacy and advanced numerical techniques, the numerical simulation techniques such as CFD became a reality and offer an effective means of quantifying the physical and chemical process in the biomass thermochemical reactors under various operating conditions within a virtual environment. The resulting accurate simulations can help to optimize the system design and operation and understand the dynamic process inside the reactors.

CFD modeling techniques are becoming widespread in the biomass thermochemical conversion area. Researchers have been using CFD to simulate and analyze the performance of thermochemical conversion equipment such as fluidized beds, fixed beds, combustion furnaces, firing boilers, rotating cones and rotary kilns. CFD programs predict not only fluid flow behavior, but also heat and mass transfer, chemical reactions (e.g. devolatilization, combustion), phase changes (e.g. vapor in drying, melting in slagging), and mechanical movement (e.g. rotating cone reactor). Compared to the experimental data, CFD model results are capable of predicting qualitative information and in many cases accurate quantitative information. CFD modeling has established itself as a powerful tool for the development of new ideas and technologies.

However, CFD modeling for biomass thermochemical conversion still face significant challenges due to the complexity of the biomass feedstock and the thermochemical process. Biomass is a mixture of hemicellulose, cellulose, lignin and minor amounts of other organics with proportion and chemical structure affected by variety. Inorganic ash is also part of the biomass composition. The complex structure makes biomass compositions pyrolyze or degrade at different rates by different mechanisms and affect each other during thermochemical process, and it makes the biomass particle feedstock has anisotropic properties in physical characterization [1]. How to deal with or simplify the complex process is a key point for the CFD simulation model. Many studies have been done on the biomass pyrolysis kinetics and the transfer and tracking of the feedstock particles, which have applied to CFD modeling and made good achievements. Simulations on reactor design, pyrolysis process, combustion systems, particle deposit and pollutant release have been performed with CFD packages.

In this paper, we attempt to summarize the current state of various CFD applications concerning the biomass thermochemical conversion process. The challenges faced by modelers using CFD in the biomass pyrolysis are also discussed.

## 2. CFD modeling principles

Computational fluid dynamics is a design and analysis tool that uses computers to simulate fluid flow, heat and mass transfer, chemical reactions, solid and fluid interaction and other related phenomena. Comparing to the physical experiment operation, CFD modeling is cost saving, timely, safe and easy to scale-up. CFD codes turn computers into a virtual laboratory and perform the equivalent “numerical experiments” conveniently providing insight, foresight and return on investment. Various numerical techniques known as direct numerical simulation (DNS), vortex dynamics and discretization methods have been employed in the solution of the CFD model equations. The most widely used numerical techniques are discretization methods mainly including finite difference (usually based on Taylor’s series, polynomial expansions), finite elements (based on calculus of variations, and the method-of-weighted-residuals) and finite volumes method (based on control-volume formulation). Finite difference techniques are rarely used in engineering flows due to the difficulties in the handling of complex geometry [2]. Finite elements are used in the commercial packages of FIDAP and POLYFLOW. Finite volumes are now the most commonly used approach in CFD code development for its ease in the understanding, programming and versatility. The most routinely used commercial codes include ANSYS FLUENT, ANSYS CFX, PHOENICS, STAR-CD and CFD2000. The available commercial CFD programmes review and the CFD performing process introduction can be found in Xia and Sun [3], Norton *et al*.[4].

### 3. CFD sub-models of Biomass Thermochemical Conversion Process

Biomass thermochemical conversion refers to the processes of biomass gasification for gaseous fuel or syngas, fast pyrolysis for liquid bio-oil, carbonization for solid carbon or combustion for heat energy. The differences among these thermal processes are determined by the operation conditions of feed properties, oxidizer (air, oxygen or steam) amount, temperature, heating rate and residence time. These conditions change the proportions of the gas, liquid and solid products. Table 1 shows the main variants of these processes [5].

### 3.1 Basic governing equations

CFD models of the thermochemical processes include description of fluid flow, heat and mass transfer, and chemical reactions. The process fundamental governing equations are the conservation laws of mass, momentum, energy and species, namely the following equations (1)–(4), respectively.

(1) $$\frac{\partial \rho }{\partial t}+\nabla •(\rho \vec{u})=S_{p}$$

(2) $$\frac{\partial (\rho \vec{u})}{\partial t}+\nabla •(\rho \vec{u}\vec{u})=-\nabla p+\nabla •(\mu \nabla \vec{u})+S_{u}$$

(3) $$\frac{\partial (\rho H)}{\partial t}+\nabla •(\rho \vec{u}H)=\nabla •(\lambda \nabla T)+S_{H}$$

(4) $$\frac{\partial (\rho Y_{i})}{\partial t}+\nabla (\rho \vec{u}Y_{i})=\nabla •(D\nabla (\rho Y_{i}))+S_{Y}+R_{f}$$

CFD enforces these conservation laws over a discretized flow domain in order to compute the systematic changes in mass, momentum and energy as fluid crosses the boundaries of each discrete region [4].

### 3.2 Thermochemical reaction submodels

The biomass thermo conversion includes complex chemical and physical processes such as vaporization, devolatilization, volatile secondary reactions, char oxidation, coupled with the transport phenomena. Many studies have been made and many models have been built to describe the process [1, 5–9].

#### 3.2.1 Devolatilization submodels

The devolatilization process begins when the biomass temperature reaches a critical level. Many biomass devolatilization models have been developed and several reviews of these models have been made [1, 6, 8]. One-step global mechanisms and semi-global multi-step mechanisms can be basically distinguished. The simplified approaches define devolatilization rates with single- or two-step Arrhenius reaction schemes.

The one-step global mechanisms can be shown as:

(5) $$\text{Biomass}\overset{k}{\to }\text{Volatiles}+\text{Char}\;\text{or}\;\text{Biomass}\overset{k}{\to }\text{Tar}(\text{Bio}-\text{oil})+\text{Gases}+\text{Char}$$

The reaction kinetic rate (k) is expressed in single-step Arrhenius fashion as *k* =*A*exp(−*E_a_* / *RT* ) , and the devolatilization rate is

(6) $$-\frac{dm_{p}}{dt}=k[m_{p}-(1-f_{v,0})m_{p,0}]$$

where *m_p_* is the biomass particle mass, *m_p,0_* is the initial particle mass, and *f_v,0_* is the initial volatile fraction [11].

For two-step Arrhenius reaction schemes, the kinetic devolatilization rate expressions of the form proposed by Kobayashi [10] are:

(7) $$k_{1}=A_{1}\text{ exp}(-E_{a}/RT)$$

(8) $$k_{2}=A_{2}\text{ exp}(-E_{a}/RT)$$

where *k*_1_ and *k*_2_ are competing rates that may control the devolatilization over different temperature ranges. The two kinetic rates are weighted to yield an expression for the devolatilization as

(9) $$\frac{m_{v}(t)}{(1-f_{w,0})m_{p,0}-m_{a}}=\int_{0}^{t}\left(\alpha_{1}k_{1}+\alpha_{2}k_{2}\right)\exp(-\int_{0}^{t}\left(k_{1}+k_{2})dt\right)dt$$

where *m_v_*(*t*) is the volatile yield up to time *t*, *m_p,0_* is the initial particle mass at injection, α_1_ and α_2_ are yield factors, *m*_*a*_ is ash content in the particle [11].

The major limitation of one-step global schemes is that they are neither able to predict the composition of volatiles nor account for various components of the virgin biomass. One-step multi-reaction schemes have been developed to address these shortcomings and can be shown as:

(10) $$\text{Biomass}(C_{x}H_{y}O_{z})\overset{k_{i}}{\to }{\text{Pr}\text{oduct}}_{i=[\text{tar},\text{char},\text{gases}(H_{2},CH_{4},CO,CO_{2,}H_{2}O,\text{etc}.)]}$$

(11) $$\text{Biomass}\;\text{Component}(i)\overset{k_{i}}{\to }\text{Volatile}+\text{Char}$$

One of the more recent developments in one-step multi-reaction schemes for biomass fuels is the use of the distributed activation energy (DAE) approach.

**Figure 1.**
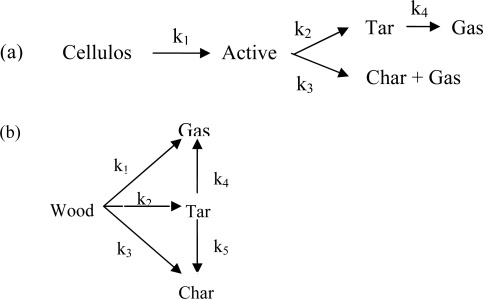
Two-stage semi-global reaction schemes for: (a) cellulose; (b) wood.

The major shortcoming of the one-step multi-reaction schemes is that they neglect secondary reactions (cracking of tar to light molecular weight volatiles). Multi-step semi-global schemes attempt to address this shortcoming of multi-reaction schemes by considering reaction routes for both primary and secondary reactions. There are many literature positions which introduced the kinetics data of these mechanisms. Figure 1. shows the two-stage semi-global reactions for cellulose and wood [6].

Another general biomass devolatilization model is developed extending the chemical percolation devolatilization (CPD) model from coal. The CPD model is extended to devolatilization of biomass major components based on the consideration of their chemical structure and its transformation under various mechanisms. The model considers multiple mechanisms, including bridge breaking and rearranging, side-chain cracking and gas release, tar distillation, and cross-linking. The same reaction scheme is applied for biomass as for coals:

(12) $$\zeta (\text{labile}\;\text{bridge})\overset{k_{b}}{\to }\zeta^{*}(\text{bridge}\;\mathrm{int}\;\text{ermedit})\begin{array}{c} \overset{k_{b}}{\to }2\delta (\text{side}\;\text{chains})\overset{k_{g}}{\to }2g_{1}(\text{light}\;\text{gases})\\ \overset{k_{c}}{\to }c(\text{char}\;\text{bridge})+2g_{2}(\text{light}\;\text{gases}) \end{array}$$

The chemical structure parameters in the original CPD model are defined directly taken from ^13^*C* nuclear magnetic resonance (NMR) measurements. The kinetic rate variables *k_b_*, *k_c_*, and *k*_δ_ are defined in Arrhenius form. The literature positions [2, 12] introduced and reviewed the CPD model and the chemical structure parameters and reaction rate expressions.

#### 3.2.2 Secondary cracking submodels

The devolatilization tar is a mixture of condensable hydrocarbons. The secondary tar crack reactions occur homogeneously in the gas phase or heterogeneously at the surface of the biomass or char particles. Tar is a complex mixture of many kinds of components and the cracking mechanism is very comprehensive. In the present study, tar cracking is considered to follow the overall reaction schemes such as:

(13) $$\text{tar}\overset{k}{\to }{\tilde{\upsilon }}_{CO}CO+{\tilde{\upsilon }}_{CO_{2}}CO_{2}+{\tilde{\upsilon }}_{H_{2}}H_{2}+{\tilde{\upsilon }}_{CH_{4}}CH_{4}+{\tilde{\upsilon }}_{\mathrm{ta}r_{\text{insert}}}tar_{\text{insert}}$$

Many experimental investigation and model studies have been done on the cracking process. The model stoichiometric coefficients and kinetics data can be found in the literatures [7, 8].

#### 3.2.3 Homogenous gas-phase reactions submodels

The biomass devolatilization and cracking gas species will react with the supplied oxidizer and with each other such as water gas shift reaction. The heat generated by exothermic reactions is important for the release of volatiles and ignition of char. The common homogeneous reactions are:

(14) $$H_{2}\frac{1}{2}O_{2}\to H_{2}O+242kJ\;/\;\text{mol}$$

(15) $$CO+\frac{1}{2}O_{2}\to CO_{2}+283kJ\;/\;\text{mol}$$

(16) $$CH_{4}+2O_{2}\to CO_{2}+2H_{2}O+35.7kJ/\text{mol}$$

(17) $$CH_{4}+H_{2}O\to CO+3H_{2}-206kJ/\text{mol}$$

(18) $$CO+H_{2}O↔CO_{2}+H_{2}+41.1kJ/\text{mol}$$

More reaction mechanisms and the kinetic parameters can be found from the literature [8].

#### 3.2.4 Heterogeneous reactions submodels

Char is the solid devolatilization residue. Heterogeneous reactions of char with the gas species such as O_2_ and H_2_O are complex processes that involve balancing the rate of mass diffusion of the oxidizing chemical species to the surface of biomass particle with the surface reaction of these species with the char. The overall rate of a char particle is determined by the oxygen diffusion to the particle surface and the rate of surface reaction, which depend on the temperature and composition of the gaseous environment and the size, porosity and temperature of the particle. The commonly simplified reactions models consider the following overall reactions:

(19) $$C+CO_{2}\to 2CO-172kJ/\text{mol}$$

(20) $$C+\frac{1}{2}O_{2}\to CO+122.9kJ\;/\;\text{mol}$$

(21) $$C+H_{2}O\to CO+H_{2}-131kJ/\text{mol}$$

The literature positions that introduced and reviewed the char surface reactions and the kinetic relationship can be found [2, 7, 13].

## 4. Additional physical models

Although Navier-Stokes equations are viewed as the basis of fluid mechanics describing the conservation laws of mass, momentum, and energy, they have a limited amount of applications in the areas of biomass thermochemical conversion. The additional processes may influence the dynamics of the thermochemical reactor system. The basic governing equations need to be strengthened with special additional physical models or assumptions to fully represent the physical process. The important additional models include turbulence models, porous media and multiphase models, heat transfer with radiation models, and mass transfer and diffusion.

### 4.1 Turbulent flow

Turbulent flows are characterized by fluctuating velocity fields primarily due to the complex geometry and/or high flow rates. Turbulence affects the heat and mass transfer and plays an essential role in some processes such as biomass gasification/pyrolysis in fluidized bed and non-premixed combustion in furnaces. The Navier-Stokes equations can be solved directly for laminar flows, but for turbulent flows the direct numerical simulation (DNS) with full solution of the transport equations at all length and time scales is too computationally expensive since the fluctuations can be of small scale and high frequency. The DNS is only restricted to simple turbulent flows with low to moderate Reynolds numbers. In the cases of high Reynolds number flows in complex geometries, a complete time-dependent solution of the instantaneous Navier-Stokes equations is beyond the nowadays computational capabilities. Hence, turbulence models are required to account for the effects of turbulence rather than simulate it directly in practical engineering applications. Two alternative methods are employed to transform the Navier-Stokes equations so that the small eddies do not have to be directly simulated: Reynolds averaging and filtering. Both methods introduce additional terms in the governing equations that must be modeled for turbulence closure.

#### 4.1.1 RANS-based models

The Reynolds-averaged Navier-Stokes (RANS) equations represent transport equations for the mean flow quantities only, with all the scales of turbulence being modeled. The RANS models are developed by dividing the instantaneous properties in the conservation equations into mean and fluctuating components, as shown as:

(22) $$\varphi =\bar{\varphi }+\varphi^{\prime }$$

The Favre-averaging (density-weighted averaging) of the flow field variables is used to account for the effects of density fluctuations due to turbulence. The classical Reynolds averaging technique brings unclosed Reynolds stress terms in the time-averaged conservation equations and need be modeled for turbulence closure. The Reynolds-averaged approach is generally adopted for practical engineering calculations.

Most common RANS models employ the Boussinesq hypothesis (eddy viscosity concept, EDC) to model the Reynolds stresses terms. The hypothesis states that an increase in turbulence can be represented by an increase in effective fluid viscosity, and that the Reynolds stresses are proportional to the mean velocity gradients via this viscosity. Models based on this hypothesis include Spalart-Allmaras, standard k-ε, RNG k-ε, Realizable k-ε, k-ω and its variants [11].

The Reynolds stress model (RSM) closes the Reynolds-averaged Navier-Stokes equations by solving transport equations for the Reynolds stresses directly, together with an equation for the dissipation rate. The RSM accounts for the effects of streamline curvature, swirl, rotation, and rapid changes in strain rate in a more rigorous manner than one-equation and two-equation models. The fidelity of RSM predictions is still limited by the closure assumptions employed. The modeling of the pressure-strain and dissipation-rate terms is particularly challenging, and often considered to be responsible for compromising the accuracy of RSM predictions. However, use of the RSM is a must when the flow features of interest are the result of anisotropy in the Reynolds stresses, for examples the cyclone flows or highly swirling flows in combustors.

#### 4.1.2 LES models

Large eddy simulation (LES) solves “filtered” transport equations by permitting direct simulation of large scale turbulent eddies. Filtering removes eddies that are smaller than the filter size, which is usually taken as the mesh size. The filtering process creates additional unknown terms that must be modeled in order to achieve closure. LES provides an accurate solution to the large scale eddies akin to DNS while the smaller eddies below the filter size are modeled. This is because the large turbulent eddies are highly anisotropic and dependent on both the mean velocity gradients and the flow region geometries, while smaller eddies possess length scales determined by the fluid viscosity and are consequently isotropic at high Reynolds numbers. LES offers an alternative method of reducing the errors caused by RANS and providing a more accurate technique for turbulence simulation. However, application of LES to biomass industrial engineering is still in its infancy for it is computational expensive [11].

### 4.2 Radiation modeling

The radiative transfer equation (RTE) for an absorbing, emitting, and scattering medium at position $\vec{r}$ in the direction $\vec{s}$ can be written as follows:

(23) dI(r→,s→)ds+(a+σs)I(r→,s→)=an2σT4π+σs4π∫04πI(r→,s→')Φ(r→,s→')dΩ'

A semi-transparent medium is considered and the refractive index is equal to unity. The optical thickness *aL* where *L* is an appropriate length scale is a good indicator of which model to use. When *aL*≫l the P-1 and Rosseland models are suitable. The P-1 model should typically be used for optical thicknesses large than 1. The Rosseland model is computationally cheaper and more efficient but should only be used for optical thicknesses larger than 3. The Discrete Ordinates model (DOM) model works across the range of optical thicknesses, but is substantially more computationally expensive than the Rosseland model [11, 14].

#### 4.2.1. Discrete Ordinates model

The Discrete Ordinates Model (DOM) solves the radiative transfer equation (RTE) for a finite number of discrete solid angles, each associated with a vector direction *s_i_* (i = 1, 2, . . . , n) fixed in the global Cartesian system, and the integrals over these directions are replaced by numerical quadratures. The DOM considers the radiative transfer equation (RTE) in the direction *s_i_* as a field equation, thus the RTE is transformed into a transport equation for radiation intensity in the spatial coordinates:

(24) ∇•(I(r→,s→)s→)+(a+σs)I(r→,s→)=an2σT4π+σs4π∫04πI(r→,s→')Φ(r→,s→')dΩ'

The standard form DOM suffers from a number of serious drawbacks, such as false scattering and ray effects. Perhaps the most serious drawback of the method is that it does not ensure conservation of radiative energy. This is a result of the fact that the standard discrete ordinates method uses simple quadrature for angular discretization. Thus, it is a logical step in the evolution of the method to move to a fully finite volume approach, in space as well as in direction. The finite volume method uses an exact integration to evaluate solid angle integrals and the method is fully conservative [11].

#### 4.2.2. P-1 model

P-1 model is the simplest formulation of the more general P-N radiation model, which is based on the expansion of the radiation intensity I into an orthogonal series of spherical harmonics. The method of spherical harmonics provides a vehicle to obtain an approximate solution of arbitrary high order (i.e. accuracy), by transforming the radiative transfer equation into a set of simultaneous partial differential equations. Using only four terms in the series solution of the respective differential equation, the following relation is obtained for the radiation flux:

(25) $$q_{r}=-\frac{1}{3(\alpha +\sigma_{s})-C\sigma_{s}}\nabla G$$

where G is the incident radiation. The problem is then much simplified since it is only necessary to find a solution for G rather than determining the direction dependent intensity. Then the following expression for *q_r_* can be directly substituted into the energy equation to account for heat sources (or sinks) due to radiation [11]:

(26) $$-\nabla q_{r}=aG-4a\sigma T^{4}$$

#### 4.2.3. Rosseland model

The Rosseland radiation model can be derived from the P-1 radiation model with some approximations. The radiative heat flux vector in a gray medium is approximated by

(27) $${\vec{q}}_{r}=-\text{Γ}\nabla G$$

The Rosseland radiation model differs from the P-1 model in that the Rosseland model assumes the intensity equal to the black-body intensity at the gas temperature. Thus,

(28) $$G=4\sigma n^{2}T^{4}$$

while the P-1 model actually calculates the transport equation for G. Substituting this value for G into Equation (27) yields

(29) $$q_{r}=-16\sigma \text{Γ}n^{2}T^{3}\nabla T.$$

This model is also called “diffusion approximation” model, since the radiation problem reduces to a simple conduction problem with strongly temperature dependent conductivity. It is important to keep in mind that the diffusion approximation is not valid near a boundary [11].

#### 4.2.4 Discrete Transfer Radiation Model

The main assumption of the Discrete Transfer Radiation Model (DTRM) is that the radiation leaving the surface element in a certain range of solid angles can be approximated by a single ray. The equation for the change of radiant intensity, *dI* , along a path, *ds* , can be written as:

(30) $$\frac{dI}{ds}+aI=\frac{a\sigma T^{4}}{\pi }$$

Here, the refractive index is assumed to be unity. The DTRM integrates Equation (30) along a series of rays emanating from boundary faces. If *a* is constant along the ray, then *I*(*s*) can be estimated as:

(31) $$I(s)=\frac{\sigma T^{4}}{\pi }(1-e^{-as})+I_{0}e^{-as}$$

The “ray tracing” technique used in the DTRM can provide a prediction of radiative heat transfer between surfaces without explicit view-factor calculations. The accuracy of the model is limited mainly by the number of rays traced and the computational grid [11].

### 4.3 Mixture fraction model

The mixture fraction model is used to present the reaction chemistry in the probability density function (PDF) method for solving turbulent-chemistry interaction. The equilibrium model is applied which assumes that the chemistry is rapid enough for chemical equilibrium to always exist at the molecular level. Basing on the simplifying assumptions, the instantaneous thermo chemical state of the fluid is related to the mixture fraction *f*. An algorithm based on the minimization of Gibbs free energy is used to compute species mole fractions from *f*. The mixture fraction *f* is defined in terms of the atomic mass fraction as:

(32) $$f=\frac{Z_{j}-Z_{j,ox}}{Z_{j,\text{fuel}}-Z_{j,ox}}$$

where *Z_j_* is the mass fraction for element *j*. The subscript *ox* and *fuel* denote the value at the oxidizer stream inlet and the fuel stream inlet respectively.

Under the assumption of equal diffusivities, the species equations can be reduced to a single equation for the mean (time-averaged) mixture fraction f̄. And the mean mixture fraction variance $\bar{f^{'2}}$ is used in the closure model describing turbulence-chemistry interactions. The transport equations for f̄ and $\bar{f^{'2}}$are:

(33) $$\frac{\partial }{\partial t}(\rho \bar{f})+\nabla •(\rho \vec{u}\bar{f})=\nabla •\left(\frac{\mu_{t}}{\sigma_{t}}\nabla \bar{f}\right)+S_{pm}$$

(34) $$\frac{\partial }{\partial t}(\rho \bar{f^{'2}})+\nabla •(\rho \vec{u}\bar{f^{'2}})=\nabla •\left(\frac{\mu_{t}}{\sigma_{t}}\nabla \bar{f^{'2}}\right)+C_{g}\mu_{t}(\nabla^{2}\bar{f})-C_{d}\rho \frac{\varepsilon }{\kappa }\bar{f^{'2}}$$

The source term *S_pm_* is due solely to transfer of mass into the gas phase from reacting sludge particles [11].

### 4.4 Porous media and two-phase model

The porous media assumption is generally used in the applications of biomass pyrolysis in fixed bed. The arrangement of biomass particles in the fixed bed forms void spaces. The devolatilization volatiles and gases through the particle voids can be described as flow through a porous media. The particle position may change during the conversion process for the devolatilization, combustion and shrinkage of biomass particles. In this process to mesh all associated geometry with a complex unstructured or body fitted system is out of both computational power and CFD algorithms levels. Therefore, the simplified porous media assumption applies Darcy’s law to present the relationship on pressure drop and volume averaged velocity caused by viscous drag:

(35) $$\nabla p=-\frac{\mu \to }{\alpha }v$$

At high flow velocities, the modification of this law provides the correction for inertial losses in the porous medium by Darcy-Forchemier equation:

(36) $$\frac{\partial p}{\partial x}=-\frac{\mu }{\alpha }v+\rho C_{F}v^{2}$$

Fluid flow, and heat and mass transfer are described in the sub-domain by the laws of conservation of mass, momentum and energy in the terms of macroscopic variables provided by the volume-averaged Navier-Stocks equations in a version of Darcy’s law. The system can be regarded as a two-phase flow [3].

### 4.5 The Lagrangian particle model

The flow in biomass fluidized bed gasifier or boilers and furnaces is a typical kind of gas-solid flow with chemical reactions. Thus hydrodynamics of the gas-solid flow can be performed based on the Eulerian–Lagrangian concept. The discrete phase method can be applied to the particle flow when the particle phase can be considered to be sufficiently dilute that the particle-particle interactions and the effects of the particle volume fraction on the gas phase can be assumed neglected. The coupling of the continuous phase and the discrete phase is important and it is solved by tracking the exchange of mass, momentum and energy.

The model computes the particle trajectory using a Lagrangian formulation which includes the inertia, hydrodynamic drag, and the force of gravity. The particle trajectory can be predicted for the *x_i_*(*i* = 1, 2, 3 for three dimension) direction in Cartesian coordinates by [11]:

(37) $$\frac{d^{2}x_{i}}{dt^{2}}=F_{D}(u_{i}-u_{p,i})+\frac{g_{x_{i}}(\rho_{p}-\rho )}{\rho_{p}}+F_{x_{i}}$$

where *F_xi_* is the additional force, *F_D_*(*u_i_* − *u_p,i_*) is the drag force per unit particle mass and

(38) $$F_{D}=\frac{18\mu }{\rho_{p}{d_{p}}^{2}}\frac{C_{D}\text{Re}}{24}$$

## 5. CFD Applications in Biomass Thermochemical Conversion Process

### 5.1 Applications in biomass gasification and pyrolysis

Biomass gasification and pyrolysis are thermally degraded processes in insufficiency or absence of air/oxygen aiming at the production of solid (charcoal), liquid (tar/bio-oil) and gaseous products. The CFD models used to describe these processes have become an important analysis and design tool to achieve the flow and temperature pattern, the products concentration contour and yields. Table 2 summarizes some of the recent studies.

**Figure 2.**
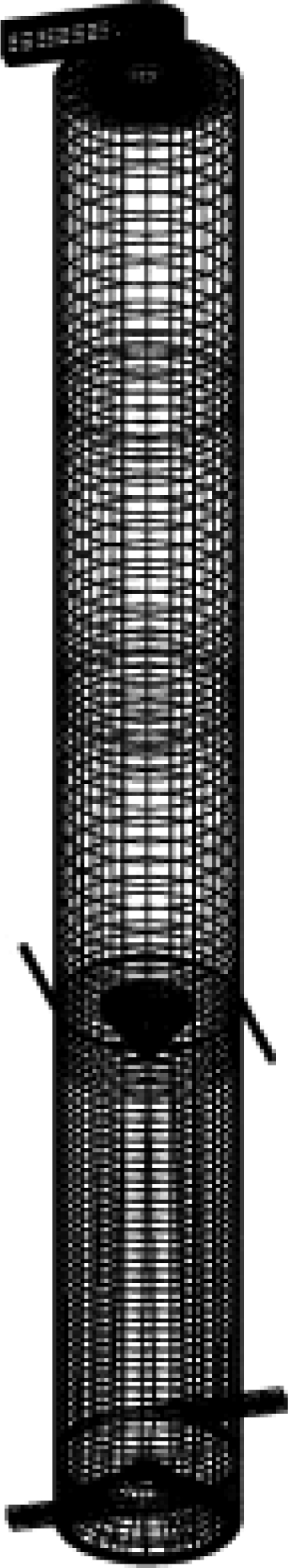
The geometry of the gasifier. The lower inlets are used to inject the biomass mixed with air, and the upper inlets are used to inject steam [15].

Fletcher *et al*. [15] developed a detailed CFD model to simulate the flow and reaction in an entrained flow biomass gasifier. The model is based on the CFX package and describes the phenomena of turbulent fluid flow, heat transfer, species transport, devolatilization, particle combustion, and gas phase chemical reactions. Biomass particulate is modeled via a Lagrangian approach as it enters the gasifier, releases its volatiles and finally undergoes gasification. Transport equations are solved for the concentration of CH4, H2, CO, CO2, H2O and O2 and heterogeneous reactions between fixed carbon and O2, CO2 and H2O are modeled. Figure 2 shows the geometry and surface mesh of the gasifier. The model provides detailed information on the gas composition and temperature at the outlet and allows different operating scenarios to be examined in an efficient manner. The initial calculations suggest that simulations to examine the effect of gasifier height and the steam flux in the upper inlets can be beneficial in process optimization. The simulation of sawdust gasification in one case gave an exit composition on a dry basis of 10% CO, 12% CO2, 20% H2 and 1.2% CH4, compared with 16% CO, 14% CO2, 10% H2, 1% CH4 measured in the experiments, the hydrogen generation was too high. The model with further validation against detailed experimental data, will aid with the design process of such gasifiers.

**Figure 3.**
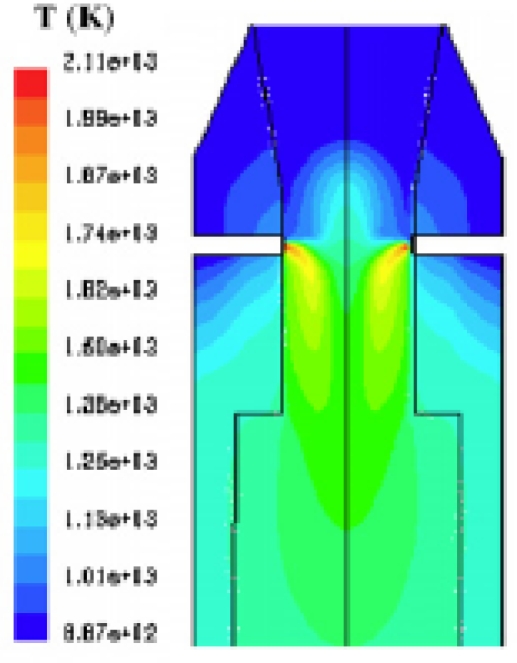
Temperature profile in the reactor [16].

**Figure 4.**
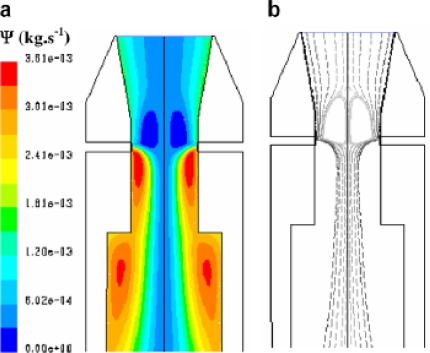
Velocity pattern in the reactor [16].

Gerun *et al*. [16] developed a 2D axisymmetric CFD model for the oxidation zone in a two-stage downdraft gasifier. The oxidation zone is crucial for tar cracking. The simulations fit satisfactorily to the experimental data regarding temperature pattern and tar concentration. Figure 3 shows the temperature profile in the reactor. The heat of reaction is released mainly close to the injector. It induces a very hot zone in this area. The stream function is shown in Figure 4a, whereas Figure 4b presents the gas pathlines in the reactor. The gas path strongly depends on the initial departure point. The strong recirculation zone is located above the air injection in the centre of the reactor. It plays a major role in air–gas mixing and thus enhances the quality of the gasification.

Table 2 lists the examples of CFD applications in biomass gasification and pyrolysis at present. The submodels used in these examples are summarized in the table.

### 5.2 Applications in biomass combustion or co-firing boilers and furnaces

The largest application of CFD models has been to power station boilers and furnaces. Many studies made in relation to coal combustion have been modified to apply to biomass combustion or co-firing. Tables 3 and 4 summarize the recent studies that apply CFD to simulate biomass combustion and co-firing boilers and furnaces. CFD modeling has established itself as a critical tool for the development of new ideas and advanced technologies. It is capable of predicting qualitative information and quantitative information to within sufficient accuracy to justify engineering design changes on commercial boiler plant.

**Figure 5.**
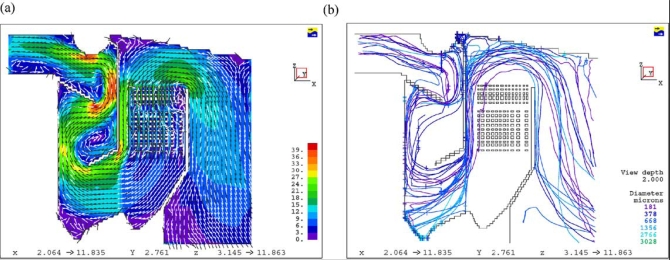
Flow simulations for the as-constructed design: (a) Gas velocity; (b) particle trajectory [24].

**Figure 6.**
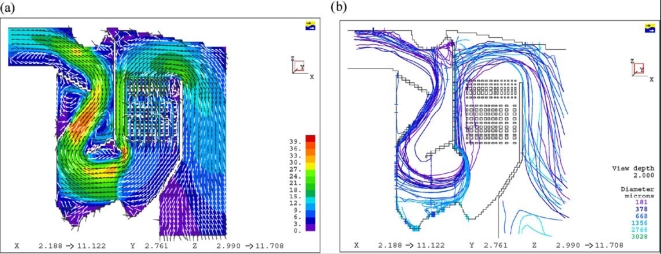
Flow simulations for the modified design: (a) Gas velocity; (b) particle trajectory [24].

Dixon *et al*. [24] summarized the CFD applications on bagasse-fired boilers in a sugar industry plant for researching the tube erosion, convection bank heat transfer, airheater corrosion, secondary air injection for furnace flame manipulation, and ignition stability and swirl burner technology. Figures 5 and 6 show a typical CFD erosion application in a tube bank for the boiler. Gas velocity contours (a) and trajectories for several particle fractions (b) are shown for the as-constructed design (Figure 5) and the modified design (Figure 6). The improvements in boiler tube erosion performance can be deduced by visual assessment alone of the predicted flow and trajectory patterns.

**Figure 7.**
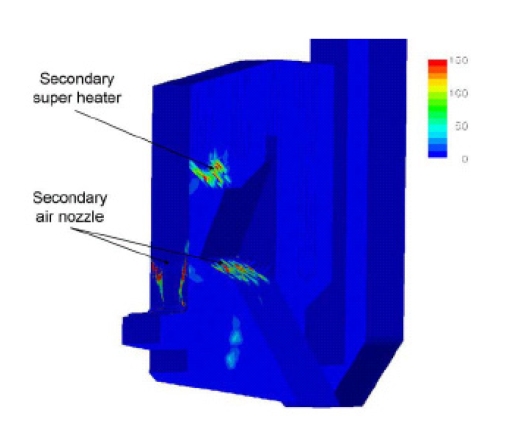
Predicted deposition mass flux in gm^−2^h^−1^ [26].

**Figure 8.**
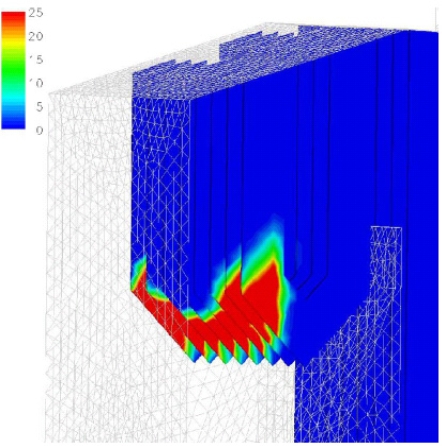
Close-up of the secondary super heater showing boundary layer controlled deposition flux in gm^−2^h^−1^. [26]

**Figure 9.**
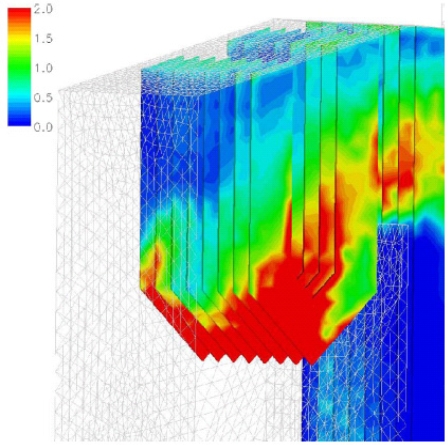
Close-up of the secondary super heater showing vapour deposition flux in gm^−2^h^−1^. [26].

Kær *et al*. [25–28] carried out CFD modeling of a 33 MW straw-fired grate boiler incorporating a standalone bed model and a commercial CFD code for gas-space computation. Figures 7, 8 and 9 show the predicted deposition mass flux of the first simulation, the boundary layer controlled deposition and the vapour deposition. He concluded that poor mixing in the furnace is a key issue leading to high emission levels and relatively high amounts of unburnt carbon in the fly ash. The model was found to correctly predict operational trends same to the boiler experiment. In the future, a significant effort will be put into further improvements and validation of the modeling concept especially with respect to the deposition velocity concept and the tube bank model.

Table 3 lists the recent studies of CFD applications in biomass combustion. The submodels used in these examples are summarized in the table.

**Figure 10.**
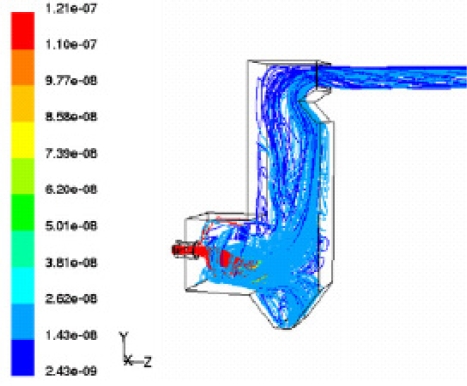
Predicted particle traces coloured by particle mass (kg) for Thoresby coal–biomass combustion cases: 0.75 mm diameter biomass particles [35].

The co-firing of coal and biomass has been advocated for a number of years as being advantageous on both an environmental and economic basis. The co-combustion of biomass as a minor component presents an interesting intermediate situation with a high reactivity solid. There are a number of commercially available CFD models, and the suitability of the sub-models available for biomass combustion is a key factor in selecting an appropriate code. Table 4 summarizes the recent CFD applications in biomass co-firing. Backreedy *et al*. [35] carried out a CFD modeling study to examine the co-firing of pulverized coal and biomass with particular regard to the burnout of the larger diameter biomass particles. The effects of the wood particle size and shape on the burnout of the combined wood and coal char were investigated. The effect of varying the devolatilization and char combustion rate constants for the biomass component in the blend was also investigated. Figure 10 shows the biomass particle tracks in the coal-biomass combustion case.

Table 4 lists the recent studies of CFD applications in biomass co-firing. The submodels used in these examples are summarized in the table.

### 5.3 Applications in the NO_x_ release

**Figure 11.**
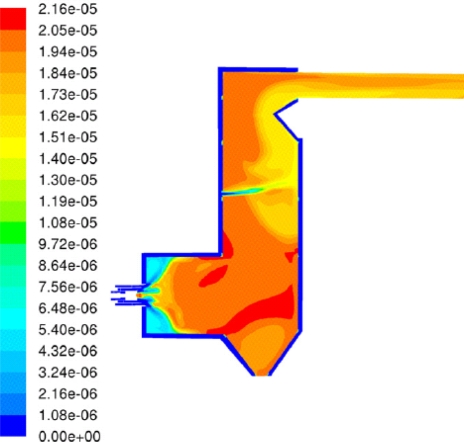
Predicted contours of potassium concentration (mol/mol) [39].

**Figure 12.**
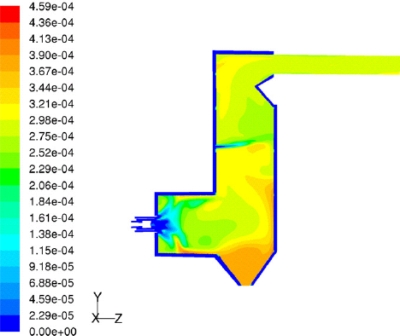
Predicted NO formation in the furnace through the NH3 route (mol/mol) [39].

In the case of biomass burner studies there is considerable interest in *NO_x_* formation and unburned carbon in ash. The literatures [39–45] described the biomass combustion and *NO_x_* formation in detail. Ma *et al*. [39] performed CFD application in a 1 MW industrial wood test furnace coupled with the potassium release and *NO_x_* formation model. The potassium release during biomass combustion is still a subject of current investigation. Ma *et al*. assume that the biomass potassium release during devolatilization rapidly forms KOH. Figure 11 shows the predicted contours of potassium concentration in the vertical symmetric plane of the furnace. Both the HCN and the *NH*_3_ route have been considered for the *NO_x_* formation and Figure 12 shows the predicted NO concentrations through *NH*_3_ route. The particle tracks and temperature distribution are also studied in this work. Good agreement between the predicted and the measured furnace temperature and concentrations of *CO*_2_ and *NO_x_* has been achieved. Table 5 summarizes the recent CFD applications in the *NO_x_* emission modeling. The submodels used in these examples are summarized in the table.

## 6. Conclusions

This paper summarized the CFD applications in biomass thermochemical conversion and system design. There is evident that CFD can be used as a powerful tool to predict biomass thermochemical processes as well as to design thermochemical reactors. CFD has played an active part in system design including analysis the distribution of products, flow, temperature, ash deposit and *NO_x_* emission. The CFD model results are satisfactory and have made good agreements with the experimental data in many cases. However, the simulations still have many approximate models as well as some assumptions. To ensure CFD simulations are more than just theoretical exercises, experimental validation is necessary to facilitate the model accuracy. With the progressing of the computing power and the development of chemical and physical models, the CFD applications in the biomass thermochemical conversion will more widely spread in the future.

## Figures and Tables

**Table 1.** Thermochemical conversion variant

Technology	Residence time	Heating rate	Temperature °C	Aim Products	Oxidizer amount
carbonation	very long (days)	low	low (~400)	charcoal	absence
fast pyrolysis	short (<2 sec)	high (>1000°C/s)	moderate (~500)	bio-oil, chemicals	limited
gasification	long	high	high (~800)	Gas, chemicals	limited
combustion	long	high	high	heat	enough

**Table 2.** CFD applications in biomass gasification and pyrolysis.

Application	Code	Dim	Aim/Outcome	Turb. Model	Extra Model	Agreement with Exp.	Authors
Entrained flow gasifier [15]	CFX4	3D	Products mass fraction distribution; temperature contours; swirl velocity distribution	Std *k* – ε RSM	Lagrangia n	Acceptable	Fletcher, D. F.
Two-stage downdraft gasifier [16]	Fluent	2D	To investigate in detail the oxidation zone; temperature profile; velocity pattern; tar conversion mechanism study	RNG *k* – ε	DOM	Satisfactory	Gerun, L.
Horizontal entrained-flow reactor [17]	Fluent	2D	Predictions of flow, temperature and conversion; sensitivity of the kinetic parameters of pulverized corn stalk fast pyrolysis	n/a	Lagrangia n	Reasonable	Xiu, S. N.
Cone calorimeter reactor [18, 19]	Code	3D	To model heat transfer and pyrolysis within dry and wet wood specimens, and the mixing and pilot ignition of the released volatiles	n/a	Porous	n/a	Yuen, R. K. K.
Moving packed bed [20]	Fluent	2D	Detailed comparisons between the combustion mode and gasification mode in a waste moving-grate furnace	Std *k* – ε	DOM	n/a	Yang, Y. B.
Entrained flow gasifier [21]	CFX	2D	To model black liquor gasification, model parameters identification and sensitivity analysis	Std *k* – ε	Lagrangia n DTRM	n/a	Marklund , M.
Downdraft gasifier [22]	Code	3D	Temperature profile, pressure drop, model parametric analysis	n/a	Porous	n/a	Sharma, A. K.
Fluidized bed flash pyrolysis [23]	Code	3D	An integrated model proposed to predict wood fast pyrolysis for bio-oil	n/a	Radiation	Good	Luo, Z. Y.

Dim=Dimension, Turb=Turbulence, Std=Standard, DOM = Discrete Ordinates Model (radiation), DTRM=Discrete Transfer Radiation Model, exp=experiment,

**Table 3.** CFD applications in biomass combustion.

Application	Code	Dim	Aim/Outcome	Turb. Model	Extra Model	Agreement with Exp.	Authors
Bagasse fired boilers[24]	Furnace	3D	Tube erosion; heat transfer Airheater corrosion; Swirl burner	Std *k* – ε	Lagrangian; porous	Acceptable	Dixon, T. F.
Straw-fired grate boiler [25–28]	CFX	3D	To provide insight into the boilers; heat transfer predictions; To predict ash deposition	RNG *k* – ε	DTRM	Good	Kær, S. K.
Combustion Furnace[29]	Fluent	3D	Particle tracks, temperature contours	Std *k* – ε	Lagrangian; DOM	n/a	Shanmukharadhya, K. S.
Waste rotary kiln incinerator [30]	Fluent	3D	To describe the processes occurring within the gaseous phase of the kiln and of the post combustion chamber	Std *k* – ε	P1	n/a	Marias, F.
Bagasse-fired furnaces [31]	Fluent	3D	To gain insight into the effect of moisture on the flame front.	*k* – ε	Lagrangian; P1	n/a	Shanmukharadhya, K. S.
Tube stove[32]	CFX-TASCf low	3D	To understand the aero-thermo-chemical behaviour of the stove operation in combustion and gasification modes	n/a	c-phase	Excellent	Dixit, C. S. B
Waste-to-energy plant[33]	Fluent FLIC		To maximize the energy recovery efficiency of waste-to-energy plants	*k* − ω	DOM	n/a	Goddar, C. D.

Dim=Dimension, Turb=Turbulence, Std=Standard, DOM = Discrete Ordinates Model (radiation), DTRM=Discrete Transfer Radiation Model , P1=P1 radiation model, exp=experiment

**Table 4.** CFD applications in biomass co-firing.

Application	Code	Dim	Aim/Outcome	Turb. Model	Extra model	Agreement with Exp.	Authors
Biomass and coal co-fired[34]	CINAR	3D	A new approach based on neural networks is proposed	*k* – ε	Radiation; Lagrangian	n/a	Abbas, T.
Co-firing[ 35]	Fluent 6.1	3D	To predict the behaviour of the biomass in the coal flame.	RNG *k* – ε	P1 FG-biomass	n/a	Backreedy, R. I.
Co-firing combustors [36]	Fluent UDF code		To develop a fragmentation subroutine applicable to Fluent via a UDF.	n/a	Lagrangian; fragmentation model	Reasonable	Syred, N.
Co-combustion boilers[37]	Fluent 6.1 MAT- LAB	3D	To optimize burner operation in conventional pulverized-coal-fired boilers	Std *k* – ε	DOM	n/a	Tan, C. K.
Biomass utility boiler[38]	Fluent 5.6	3D	To examine the impact of the large aspect ratio of biomass particles on carbon burnout in cofiring switchgrass/coal.	Std *k* – ε	Lagrangian; DOM	n/a	Gera, D.

Dim=Dimension, Turb=Turbulence, Std=Standard, DOM = Discrete Ordinates Model (radiation); P1=P1 radiation model, exp=experiment

**Table 5.** CFD applications in *NO_x_* formation of biomass thermochemical conversion.

Application	Code	Dim	Aim/Outcome	Turb, Model	Extra Model	Agreement with Exp.	Authors
Test furnace [39]	Code	3D	Particle tracks, temperature contours, NO formation, potassium concentration	RNG *k* – ε	Lagrangian; P1; radiation; *NO_x_* Formation; potassium release;	Good	Ma, L.
Combustion chamber [40]	Fluent 5.5	3D	Prediction of gaseous emission	SST- *k* – ω	Lagrangian; DTRM; *NO_x_*- model	Good	Miltner, M.
Pilot down-fired combustor [41]	Fluent 5.0	3D	To describe the processes occurring within the gaseous phase of the kiln and of the post combustion chamber	*k* – ε	P1; Lagrangian; *NO_x_* module	n/a	Zarnes-cu, V.
Fluidized beds [42]	Fluent 6.2	3D	To compare the performance of five global ammonia chemistry mechanisms in full-scale boiler CFD modeling.	Std *k* – ε	DOM; Global Ammonia Chemistry Mechanism s	Well under special conditions	Saario, A.
Biomass combustion [43]	Code	1D	Comparisons of the Validity of Different Simplified NH3-Oxidation Mechanisms for Combustion of Biomass	n/a	Ammonia oxidation mechanisms	n/a	Norstrom, T.
Wood stove [44]	Spider	2D	To model nitric-oxide formation from fuel-bound nitrogen in biomass turbulent non-premixed flames.	Std *k* – ε	DTRM	n/a	Weydahl, T.
Bagasse-fired boiler [45]	Furnace	3D	To apply conditional moment closure (CMC) in a to obtain predictions of CO and NO in the flue gas.	Std *k* – ε	Lagrangian; DTRM; PDF; conditional moment closure equation	Reasonable	Rogerson, J. W.

Dim=Dimension, Turb=Turbulence, Std=Standard, DOM = Discrete Ordinates Model (radiation); DTRM=Discrete Transfer Radiation Model, P1=P1 radiation model, PDF= Probability Density Function, exp=experiment.
